# Digital phenotyping in young breast cancer patients treated with neoadjuvant chemotherapy (the NeoFit Trial): protocol for a national, multicenter single-arm trial

**DOI:** 10.1186/s12885-022-09608-y

**Published:** 2022-05-04

**Authors:** Lidia Delrieu, Anne-Sophie Hamy, Florence Coussy, Amyn Kassara, Bernard Asselain, Juliana Antero, Paul De Villèle, Elise Dumas, Nicolas Forstmann, Julien Guérin, Judicael Hotton, Christelle Jouannaud, Maud Milder, Armand Leopold, Adrien Sedeaud, Pauline Soibinet, Jean-François Toussaint, Vincent Vercamer, Enora Laas, Fabien Reyal

**Affiliations:** 1 Residual Tumor & Response To Treatment Laboratory, RT2Lab, Translational Research Department, INSERM, U932 Immunity and Cancer, Institut Curie, University Paris, Paris, France; 2 Department of Medical Oncology, Institut Curie, University Paris, Paris, France; 3 GINECO Group, Paris, France; 4 Institute for Biomedical and Epidemiological Research in Sport, France University, EA7329 Paris, France; 5 Institut National du Sport de L’Expertise Et de La Performance, INSEP, Paris, France; 6 Withings, Issy-les-Moulineaux, Paris, France; 7 Data Office, Institut Curie, Paris, France; 8 Department of Surgical Oncology, Institut de Cancérologie Jean-Godinot, Reims, France; 9 Department of Medical Oncology, Institut de Cancérologie Jean-Godinot, Reims, France; 10 Center for Sports Medicine Research, Hôtel-Dieu, Publics Assistance Hospitals of Paris, Paris, France; 11 Department of Surgical Oncology, Institut Curie, University Paris, Paris, France

**Keywords:** Breast cancer, Neoadjuvant, Activity trackers, Digital, Prevention

## Abstract

**Background:**

Breast cancer (BC) has particular characteristics in young women, with diagnosis at more advanced stages, a poorer prognosis and highly aggressive tumors. In NeoFit, we will use an activity tracker to identify and describe various digital profiles (heart rate, physical activity, and sleep patterns) in women below the age of 45 years on neoadjuvant chemotherapy for BC.

**Methods:**

NeoFit is a prospective, national, multicenter, single-arm open-label study. It will include 300 women below the age of 45 years treated with neoadjuvant chemotherapy for BC. Participants will be asked to wear a Withing Steel HR activity tracker round the clock for 12 months. The principal assessments will be performed at baseline, at the end of neoadjuvant chemotherapy and at 12 months. We will evaluate clinical parameters, such as toxicity and the efficacy of chemotherapy, together with quality of life, fatigue, and parameters relating to lifestyle and physical activity. The women will complete REDCap form questionnaires via a secure internet link.

**Discussion:**

In this study, the use of an activity tracker will enable us to visualize changes in the lifestyle of young women on neoadjuvant chemotherapy for BC, over the course of a one-year period. This exploratory study will provide crucial insight into the digital phenotypes of young BC patients on neoadjuvant chemotherapy and the relationship between these phenotypes and the toxicity and efficacy of treatment. This trial will pave the way for interventional studies involving sleep and physical activity interventions.

**Trial registration:**

Clinicaltrials.gov identifier: NCT05011721. Registration date: 18/08/2021.

## Background

About 5% of all breast cancers (BC) in France affect women under the age of 45 years [1]. Between 2500 and 3000 new cases of BC are detected annually in women of this age group, and these tumors are associated with a higher mortality than those in older women [2, 3]. The tumors of younger women present particular characteristics: (i) they are diagnosed at more advanced stages; (ii) aggressive cancers and genetically determined forms are overrepresented; (iii) and prognosis is poor [4, 5].

Neoadjuvant chemotherapy (NAC), is currently used for the treatment of aggressive breast carcinomas that are nonetheless operable [6], to increase conservative breast surgery rates [7]. Neoadjuvant chemotherapy has many advantages such as a less extensive breast and axillary surgery and a monitoring of the treatment response [8, 9]. Pathological complete response (pCR) has been reported to occur in about 20–60% of patients on NAC, and is associated with better survival outcomes [10–13]. NAC is increasingly prescribed notably in triple negative BC [14, 15].

BC patients may experience long- and short-term complications and sequelae due to treatment or the disease itself, including alterations to quality of life, body composition, physical fitness and physical activity levels, heart rate, sleep and fatigue [16–20]. Lower levels of physical activity have also been reported to have a negative effect on quality of life and the risk of cancer recurrence [21–25]. A lack of physical activity is the fourth leading risk factor for death worldwide, including about 21% of breast cancer deaths [26]. BC patients often decrease their levels of physical activity at diagnosis [25] and during treatment, despite the known benefits of physical activity for the physical, biological, clinical and psychological states of the patient clearly demonstrated in tertiary prevention studies[27]. However, the effects of lifestyle, including the level of physical activity, remain incompletely understood outside the domain of cancer care.

Digital health involves the collection of large amounts of data with various devices, including activity trackers and mobile phones [28]. Activity trackers can be used to evaluate the number of steps taken each day, the distance covered, energy expenditure, heart rate and the quality and duration of sleep [29]. Digital phenotyping is defined as the use of data from personal digital devices, including smartphones in particular, for instantaneous quantification of the human phenotype at individual level in situ [30]. This approach provides longitudinal data concerning the everyday activities of patients in a continuous and ecological manner. The combination of digital phenotyping data, clinical data and patient-reported outcomes can be used to identify patterns of behavior in patients; these patterns can then be used as the basis for the optimization of patient care through precision, predictive and personalized medicine [30–34].

The single-arm NeoFit trial will be an interventional single-arm study with the primary objective of describing and identifying digital profiles (physical activity, sleep and heart rate) in young BC patients (below 45 years of age) on NAC. Its secondary objectives will be: 1) to compare the digital profiles of participants according to clinical and sociodemographic variables, 2) to analyze the effects of digital profiles on tumor response, immune infiltrate, treatment toxicity, surgical complications, quality of life and fatigue, 3) to develop models for predicting chemotherapy response, toxicity, fatigue and changes in quality of life during treatment.

## Methods

### Study design

**Fig. 1:**
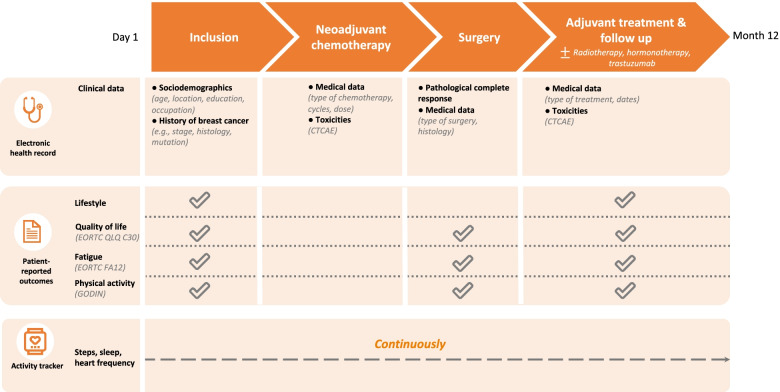
Participant flow chart for the NeoFit study, France (original flow chart)

The NeoFit study is a prospective, national, multicenter, single-arm, open-label study promoted by the Institut Curie (Paris, France) (Fig. 1). It will enroll 300 participants from two comprehensive cancer care centers in France, beginning in September 2021 during a one-year period.

This trial protocol conforms to the rules of the Declaration of Helsinki and was approved by the appropriate French ethics committee CPP Est I (ID RCB: 2021-A00268-33). The study database has been registered with the French national data protection authority (CNIL) (reference number: reference: 2,220,932 v 0). The study has been registered with http://www.clinicaltrials.gov (NCT number: NCT05011721, registration date: 18/08/2021). Written informed consent will be obtained from each participant.

### Study population

Participants meeting the following criteria will be included:

- 1) Female,
- 2) Between the ages of 18 and 45 years,
- 3) Histologically confirmed BC, whatever its histological subtype (hormone receptor-positive (RH +), negative (RH-), with (*HER2* +) or without (*HER2-*) *HER2* overexpression, or triple-negative),
- 4) NAC scheduled (up to the first day of neoadjuvant chemotherapy),
- 5) Eastern Cooperative Oncology Group Performance status ≤ 2,
- 6) Patient willing to participate for the entire duration of the study,
- 7) Patient able to use a compatible smartphone or computer tablet to download the Withings Health Mate application (from iOS 10 and Android 5.0 or above),
- 8) Patient with Internet access,
- 9) Patient with valid health insurance,
- 10) Patient able to read, write and understand French.

Patients will be considered ineligible if they meet any of the following criteria:

- 1) Metastases present at BC diagnosis,
- 2) Bilateral BC,
- 3) Multifocal BC,
- 4) History of cancer other than basal cell skin lesions and cervical dysplasia,
- 5) Patient pregnant or breastfeeding,
- 6) Impossibility of following the patient throughout the study for medical, social, family, geographic or psychological reasons,
- 7) Patient incarcerated or under a supervision order.

### Recruitment

Eligible individuals will be invited to participate in the study at the time of NAC prescription. Investigators will check the eligibility criteria at the treatment initiation consultation, explaining the objectives of the study and the way in which it will be performed to the patient. After sufficient time for reflection, patients agreeing to participate will be asked to provide written informed consent.

### Setting up the activity tracker and explaining its use

Participants will be asked to wear a Withings Steel HR activity tracker (Withings, Issy-les-Moulineaux, France) round the clock, throughout the study period. The Withings Health Mate mobile phone application will first be downloaded onto a smartphone or tablet at baseline, and the clinical research assistant will instruct the participant in the use of the activity tracker. The participant will then be asked to accept and activate the sharing of data collected with the dedicated “NeoFit” study server at Institut Curie. Participants will be asked to synchronize the activity tracker regularly (ideally daily) via Bluetooth with the Withings Health Mate application, for automatic data transfer to the secure "NeoFit" space. A clinical research assistant will optimize follow-up during the course of the study, via a platform checking the downloading of data from the activity tracker determining whether questionnaires have been completed within the time limit.

### Evaluations

#### Modes of evaluation

All participants will attend three study consultations when attending the participating hospital center for their regular oncology consultation: an initial inclusion assessment (before the first day of neoadjuvant chemotherapy), a second assessment at the end of neoadjuvant chemotherapy, and a third assessment 12 months after inclusion (M12).

The activity tracker will collect information continuously via the Withings Health Mate application. At the end of the study, participants will be allowed to keep the activity tracker, with a transfer of the corresponding account to their own name.

Patient-reported outcomes will be evaluated by the answers provided on the questionnaires completed by all participants at the various time points. Patients will be able to assess the questionnaires directly via the REDCap Internet link of the NeoFit study, with a computer, smartphone or computer tablet. Each time that a new questionnaire requiring completion is posted online, the participants will be notified by e-mail and asked to complete it. If they encounter any problems completing the questionnaire, they will be able to contact the clinical research assistant to ask for help.

### Data collection

A complete data collection schedule is provided in Fig. 1. Missing data will be limited by the checking of the NeoFit professional dashboard by the clinical research assistant, to ensure that questionnaires are completed on time.

#### Clinical data

The clinical research assistant will collect clinical data from the electronic medical records of the participants. The data collected will include information already analyzed in the care context and concerning disease status, neoadjuvant treatment, surgical data for biopsy and tumor specimens, adjuvant treatment and cancer progression. Toxicity data will be collected throughout the study. Toxicity will be defined according to the National Cancer Institute’s Common Terminology Criteria for Adverse Events (NCI-CTCAE) v5.0, as the occurrence of severe toxicity of at least grade 3.

#### Lifestyle and sociodemographic data

Lifestyle and sociodemographic data will also be collected. We will record the socioprofessional status of participants, their alcohol and tobacco consumption, eating habits, and the distance between the patient’s home and the center (baseline and Month 12), via a non-validated self-administered questionnaire with an estimated completion time of 10 min.

#### Quality of life

Quality of life will be assessed with the European Organisation for Research and Treatment of Cancer Quality of Life Questionnaire (EORTC QLQ-C30) version 3, which was validated in 2000 [35]. This multidimensional questionnaire has been validated for use with cancer patients. It contains 30 items assessing five functional domains (physical, role, emotional, cognitive, and social), one overall quality-of-life domain, three symptom domains (pain, fatigue and nausea), and six individual items (dyspnea, insomnia, anorexia, diarrhea, constipation, and financial impact). Responses will be on a Likert scale ranging from "not at all" to "very much" and from "very poor" to "excellent" for the overall quality-of-life questions only. Scores will be standardized on a scale of 0 to 100. Higher scores will correspond to better functioning, a better overall quality of life and more symptoms. Mean completion time for this questionnaire has been estimated at 10 min.

#### Fatigue

Fatigue will be evaluated with the EORTC QLQ-FA12 version 1 module, which was validated for cancer-related fatigue in 2017 [36]. EORTC QLQ-FA12 contains 12 items assessing the physical, cognitive, and emotional domains of cancer-related fatigue. Participants will complete a four-point Likert-scale questionnaire, with responses ranging from "not at all" to "very much".

Scores will be standardized on a scale of 0 to 100, with higher scores suggesting a greater degree of fatigue. This questionnaire has an estimated completion time of five minutes.

#### Physical activity level

Physical activity level will be assessed with the Godin Leisure-Time Exercise Questionnaire (GSLTPAQ) version 1, validated for women with BC in 2015 [37, 38]. This validated self-administered questionnaire contains three main questions on the frequency of low-intensity (e.g., easy walking), moderate-intensity (e.g., brisk walking), and high-intensity (e.g., jogging) physical activity for at least 15 min in a typical week. The frequency of low-intensity exercise is then multiplied by three, that for moderate-intensity exercise is multiplied by five and that for high-intensity exercise is multiplied by nine and the various scores are then added together to obtain a total score. This total score identifies three categories of patients on the basis of physical activity levels: highly active (≥ 24 units), moderately active (14 to 23) and with low levels of activity (< 14 units). Completion time for this questionnaire has been estimated at three minutes.

#### Activity tracker

The activity tracker will record daily step counts, heart rate at 10-min or one-second (if the patient manually activates the workout mode) intervals, sleep parameters (total sleep duration, duration of the deep and light phases of sleep, number of awakenings and their duration, and sleep quality according to a score developed by Withings) and other physical activities recorded automatically or declared by the participant [39–44].

### Data security

#### REDCap eCRF

The REDCap database is accessible via a protected Internet interface. User authentication is based on the directory of Institut Curie employees. On connection to the database, users are asked to confirm their identity using the credentials of their institutional accounts.

REDCap has a strong authentication system, based on a "two-factor" authentication principle, with a validation code transmitted by SMS or e-mail at each connection. An audit trail system is used to trace any changes. Additions, deletions or modifications to the data are recorded, along with the identity of their authors.

The REDCap database is hosted on the institutional MySQL database cluster managed by the IT department. Mirror databases are housed on two separate servers: a master server and a backup server in case of incidents. The database is backed up daily and stored in dedicated spaces.

#### Withings Application Programming Interface (API)

A free guide to the use of the Withings API is available online (https://developer.withings.com). The data will be stored in a databased dedicated to the NeoFit project and will be accessible to only a few authorized members of the Data Office, for maintenance and automatic data feeding purposes, and to identified members of the project team for data analysis. Access to this database will be granted via dedicated user accounts.

The Withings storage database is fed by an automatic routine, with users unable to perform manual interventions. None of the data are deleted or modified. Data can only be added, by the retrieval, at a predetermined frequency, of new measurements from the activity tracker. The Withings storage database is hosted on a dedicated instance within the data infrastructure, a virtualized infrastructure distributed over 12 physical servers. It is therefore resilient to failure and is backed up daily.

### Statistical analysis

#### Sample size

 As the main objective of the study is descriptive, there is no need to define a minimum number of subjects. For model validation, we will divide our population into two groups: i) a training sample (*n* = 150) and ii) a validation sample (*n* = 100). The accuracy of the model for 150 subjects will therefore be about 8%.

$$CI=2\times \surd \frac{p\times q}{n}$$

We hypothesize that 10% of the participants may be lost to follow-up. In the context of this study, that would mean not completing the questionnaires despite reminders, or not wearing the activity trackers. The inclusion of 300 participants is therefore expected to result in the collection of at least 250 completed questionnaires and 250 activity tracker datasets.

#### Statistical methods

Qualitative variables, expressed as percentages, will be used for the descriptive analysis. The number of missing data will be recorded to check for dependence on study variables and to describe the individuals lost to follow-up during the study. Quantitative data will be described by means or medians, standard deviation, minimum and maximum values. Individual trajectories may also be plotted graphically.

For the primary endpoint analysis, we will use mixed models with latent classes to identify different digital phenotypes. The use of mixed models will make it possible to analyze repeat data for the population, and to determine an average profile or trajectory for the whole population. The choice of shape for this trajectory will be determined by testing (linear, quadratic, cubic or spline shape). The optimal number of classes will be determined a posteriori*,* based on a set of statistical and clinical criteria. The most widely used statistical criterion is the "Bayesian information criterion" (BIC), for which the likelihood of the model decreases with increasing complexity. The BIC, has been shown to outperform other, less strict criteria in simulations. The number of trajectories will also be determined on the basis of clinical interpretation (e.g. whether it is worthwhile retaining classes with very few members).

For the secondary endpoint analysis, the variables will be described, by trajectory, once the classes have been defined. We will compare means (Student's *t*-test), or categorical variables (chi^2^ tests). We will also perform multinomial logistic regression analyses with univariate and multivariate models, to determine the probability of belonging to a given class relative to the corresponding reference class. Time series forecasting will be used to predict events from a series of previous observations. This will make it possible to predict physiological changes in the participant based on her care trajectory and a series of observations of her physiological state, a potentially valuable indicator of the patient’s response to neoadjuvant treatment.

No imputation procedure will be required for missing data as we will use a maximum likelihood approach. Such approaches are robust to noninformative missing data, and a joint model will be used to take informative missing data into account, if necessary.

In terms of the development of algorithms and prediction models, we will:

- i) Compare the clinical data to a historical Institut Curie database containing data for patients receiving neoadjuvant treatment for BC collected between 2002 and 2011,
- ii) Compare the characteristics of our population with those of worldwide users of Withings Steel HR activity trackers of the same age and with a similar BMI. However, we will have no access to the medical histories of the other users,
- iii) Develop models for predicting treatment efficacy and toxicity. We will use the clinical data (personal information, pathological variables and treatment-related variables) and digital phenotype of the BC patient. Treatment toxicity will be evaluated with questionnaires based on the CTCAE criteria. Chemotherapy efficacy will be assessed by evaluating pathological complete response rates and determining the number of nodes sampled after NAC. We will train the model on 150 patients and validate it on the remaining 100 patients.
- iv) Develop models for predicting fatigue and changes in quality of life during treatment. We aim to develop a statistical model for detecting correlations between treatment-related variables (e.g. drugs taken, number of cycles and their dates) and periods of fatigue or deterioration of quality of life characterized by abnormal changes in physical activity. A training/validation strategy similar to that described in (iii) will be used.
- We will start by constructing the classification tool, which we will then validate before performing comparative studies based on the resulting classification.

Statistical analyses will be performed with R software.

### Data monitoring

The database for clinical data will be created with REDCap® software. It will have secure access (personal ID and password required), with several security levels, depending on the role of the investigator. All questionnaires will be completed on an eCRF and merged with the clinical database and the activity tracker data at the end of the study. The trial steering committee will be responsible for data monitoring, including overall project supervision, the monitoring of progress, providing advice on scientific credibility and the appropriate running of the project, and ensuring its integrity. The clinical research assistant will check all consent forms, ensure compliance with the established protocol and procedures, and will verify the quality of the data in the eCRF monthly with the NeoFit professional dashboard.

### Dissemination

Digital phenotyping can provide detailed knowledge about the non-healthcare aspects of this population, to facilitate the adjustment of prevention strategies. Specific groups of women will be identified based on the huge amounts of individual data collected by the activity tracker for physical activity, heart rate and sleep. This will make it possible to develop prevention programs based on the specific characteristics of the participants. Ultimately, this study will provide public decision-makers with high-quality scientific data to guide policies for promoting healthy behavior. The findings of the NeoFit trial will be disseminated via peer-reviewed publications and conference presentations. The datasets generated and/or analysed during the current study will not publicly available due to personal health data but will be available from the corresponding author on reasonable request at the end of the study.

## Discussion

The particular psychological and physical issues faced by young women with BC require specific management [3]. These women are at greater risk of psychological distress than older women, particularly in the context of chemotherapy [3, 45–47]. They also tend to decrease their levels of physical activity immediately after being diagnosed with BC [3]. The ESO-ESMO 4^th^ International Consensus Guidelines for Breast Cancer in Young Women recommend encouraging patients to engage in regular aerobic physical activity and providing patients with appropriate supportive care for dealing with potential long-term and late sequelae [3]. However, little is known about the potential contribution of activity trackers to this support.

Innovative connected tools effectively promote good health management and are generally well accepted by BC patients [48]. A systematic review published in 2019 explored the contribution of digital health to improving quality of life in breast cancer patients [48]. Despite differences between the 24 studies included in terms of both the tools used and study quality, it was clear that patients readily accepted and were satisfied with these new technologies. These tools can help to promote healthy lifestyles, and can overcome geographic and financial barriers, thereby reducing unnecessary costs and improving care quality [49]. Furthermore, young women with BC are particularly avid users of connected technologies, with a major presence on social networks. They are interested in new technologies, including those for electronic communication and information sharing, and search engines [50, 51]. The Precision Medicine Initiative Working Group highlighted the challenges ahead in its report, and pointed out that data from sensors and software applications can add to self-reported information about lifestyle and environment, providing researchers with a clearer picture of the contribution of these factors, which have proved difficult to capture accurately [52, 53].

Digital phenotyping approaches facilitate rapid data collection from diverse sources during patient care; they are, thus, having a revolutionary effect on clinical medicine [49, 54]. They shed light on the environment in which the patient operates, outside of the hospital care context and across the continuum of BC care, thereby making it possible to improve personalized care. It is essential to identify the needs of young BC patients and to refocus care on the patient and her living environment, rather than just the disease, in a shift towards a more more holistic form of medicine. The huge amounts of data generated by connected devices are making it possible to develop predictive models and to forecast clinical events or complications, with consequences for the actions of doctors [49, 55].

The challenges facing us in the near future include the development of methods for analyzing the massive amounts of data generated by such devices [56, 57]. Wearable devices can be used to collect activity data unobtrusively and cheaply, in the patient’s everyday environment. Although it is difficult to capture the physical activity level before the BC diagnosis, data generated by the activity trackers will allow us to describe precisely what happens during the treatment continuum. New methods and algorithms are emerging for optimizing the exploitation of the massive amounts of digital data available, but further studies are required. Digital data also raise ethical issues, concerning the site of data storage, data sharing, and the integration of these data into the patient’s care continuum. The transformation of unstructured information into structured, understandable, and transmittable information is a key issue that is providing highly challenging. The preliminary results of this descriptive study will allow the development of future randomized studies to test preventive interventions such as physical activity in this population.

In conclusion, the NeoFit Trial will help us to develop a comprehensive, integrative view of patients, the characteristics of their tumors, digital phenotyping data, treatment toxicity and efficacy, with the ultimate goal of developing actionable perspectives for increasing cure rates.

## Data Availability

Data sharing is not applicable to this protocol article, as no datasets have yet been generated or analyzed.

## Abbreviations

BC: Breast cancer
CTCAE: Common terminology criteria for adverse events
NAC: Neoadjuvant chemotherapy
TILs: Tumor-infiltrating lymphocytes
